# *Lactobacillus helveticus* UA881 Improves Body Composition, Lipid Profiles, and Gut Microbiota in Overweight Adults: A Randomized Double-Blind Placebo-Controlled Trial

**DOI:** 10.3390/biomedicines14020276

**Published:** 2026-01-26

**Authors:** Yu-Wei Chang, Yin-Chin Liu, Pin-Chao Huang, Shao-Yu Lee, Meei-Yn Lin, Chin-Lin Hsu

**Affiliations:** 1 Department of Medical Laboratory and Biotechnology, Chung Shan Medical University, Taichung 402306, Taiwan; golden3p@csmu.edu.tw; 2 Department of Clinical Laboratory, Chung Shan Medical University, Taichung 402306, Taiwan; 3 Department of Nutrition, Chung Shan Medical University, Taichung 402306, Taiwan; sm051435123@gmail.com; 4 Percheron Bioceutical Research Center, Taichung 407612, Taiwan; stanley.huang@nutrarex.com.tw (P.-C.H.); shaoyu.lee@nutrarex.com.tw (S.-Y.L.); 5 Department of Food Science and Biotechnology, National Chung Hsing University, Taichung 402202, Taiwan; 6 Department of Nutrition, Chung Shan Medical University Hospital, Taichung 402306, Taiwan

**Keywords:** metabolic health, probiotics, *Lactobacillus helveticus* (UA881), gut microbiota

## Abstract

**Background:** Overweight and metabolic disorders are strongly associated with gut microbiota dysbiosis. Probiotics represent a safe dietary strategy to improve metabolic health, although strain-specific effects remain unclear. This study evaluated the metabolic and gut microbiota-modulating effects of *Lactobacillus helveticus* (UA881) in overweight adults. **Methods:** In a randomized, double-blind, placebo-controlled trial, 50 overweight adults (Body mass index, BMI 25–27 kg/m^2^) were assigned to receive UA881 (5 × 10^9^ CFU/day) or placebo for 28 days. Anthropometric parameters, body composition, serum biochemical markers, inflammatory cytokines, fecal short-chain fatty acids (SCFAs), and gut microbiota composition (16S rRNA sequencing) were assessed at baseline and after 28 days. Statistical analyses included paired *t*-tests and ANCOVA adjusted for baseline values. **Results:** After 28 days of supplementation, UA881 significantly reduced body weight, BMI, and body fat mass. The primary endpoint, serum triglycerides, was significantly decreased, and the increases in uric acid, total cholesterol, and Low density lipoprotein-cholesterol (LDL-C) observed in the placebo group were attenuated. No significant changes were observed in interleukin-6 (IL-6) or tumor necrosis factor-α (TNF-α) levels. Fecal butanoic acid showed an increasing trend, and gut microbiota alpha diversity was significantly improved. At the genus level, *Anaerostipes* and *Blautia* were enriched, while *Collinsella* was reduced. **Conclusions:** A 28-day supplementation with *L. helveticus* UA881 (5 × 10^9^ CFU/day) improved body composition and lipid-related metabolic parameters and favorably modulated gut microbiota composition in overweight adults, supporting its potential as a probiotic candidate for metabolic health.

## 1. Introduction

The global prevalence of overweight and metabolic syndrome has risen dramatically in recent decades, posing a major challenge to public health [1]. Obesity is strongly associated with comorbidities such as type 2 diabetes mellitus, cardiovascular disease, non-alcoholic fatty liver disease, and certain cancers, all of which contribute to increased morbidity and mortality worldwide [2,3]. The World Health Organization has projected that the prevalence of overweight will continue to increase, particularly in Asia, where lifestyle changes and urbanization have accelerated dietary shifts toward high-calorie, low-fiber foods [4]. Although pharmacological therapy and bariatric surgery are available for overweight management, their application is often restricted by high cost, limited accessibility, safety issues, and poor patient compliance. Thus, there is a critical need for safe, accessible, and effective strategies to improve metabolic health in at-risk populations.

Growing evidence has highlighted the role of the gut microbiota as a key regulator of host metabolism [5,6]. The human gastrointestinal tract harbors trillions of microorganisms that interact with dietary components and host physiology, influencing energy harvest, lipid metabolism, and systemic inflammation [6,7]. Dysbiosis, or an imbalance in gut microbial composition, has been linked to overweight, insulin resistance, and atherosclerosis [8]. Mechanistically, the gut microbiota contributes to host metabolism through the production of short-chain fatty acids (SCFAs) such as acetate, propionate, and butyrate, which can regulate energy balance, gut barrier integrity, and inflammatory pathways [9]. Conversely, certain microbial taxa, including *Collinsella* and other opportunistic species, have been associated with unfavorable metabolic profiles and systemic inflammation [10]. These observations suggest that strategies targeting the gut microbiota may hold promise in modulating metabolic disease risk.

Probiotics are defined as live microorganisms that confer health benefits to the host when administered in appropriate amounts and may serve as an effective complementary strategy. Various probiotic strains, particularly those from the genera *Lactobacillus* and *Bifidobacterium*, have been shown in animal and human studies to influence weight management, lipid metabolism, and inflammatory responses [11,12]. Clinical trials have reported that probiotics can reduce body mass index (BMI), serum triglycerides, and total cholesterol levels, while improving markers of gut health [13,14]. However, findings remain inconsistent, with some studies reporting modest or no effects [15,16]. These discrepancies may reflect differences in probiotic strains, dosages, intervention durations, and study populations. Furthermore, many trials have focused on short-term outcomes, and strain-specific effects remain poorly characterized [17]. Despite these limitations, probiotics are widely recognized as safe and accessible dietary interventions, making them attractive candidates for managing overweight-related metabolic dysfunction.

In addition to clinical outcomes, probiotics may exert their effects through modulation of gut microbial diversity and community composition. Increased microbial diversity has generally been associated with improved metabolic health, while low diversity is often linked to obesity and inflammation [18]. Specific genera, such as *Anaerostipes* and *Blautia*, are known producers of butyrate, an SCFA with anti-inflammatory and metabolic benefits, whereas *Collinsella* has been correlated with adverse metabolic profiles [10,19,20]. Understanding how probiotic supplementation shifts the balance between beneficial and harmful taxa may provide important insights into their mechanisms of action.

Against this background, the present study was designed to investigate the metabolic effects of UA881, a novel probiotic formulation. UA881 is a probiotic strain originally isolated from human breast milk. It was selected for the present study based on preliminary pre-clinical screening, which suggested potential roles in purine-related metabolism, antioxidant capacity, and intestinal barrier support. These biological characteristics provided the rationale for exploring the effects of UA881 supplementation on metabolic health outcomes in overweight adults. Unlike many earlier studies that examined general probiotic supplementation, we specifically tested whether UA881 could improve metabolic outcomes in overweight individuals. We hypothesized that UA881 supplementation would lead to improvements in body characteristics and serum lipid profiles, without inducing pro-inflammatory responses, and that these effects would be mediated in part through alterations in gut microbial composition and SCFA production. To test this hypothesis, we conducted a randomized, double-blind, placebo-controlled human trial in which participants received either UA881 or a placebo for 28 days. Clinical outcomes included anthropometric measures (weight, BMI, body fat distribution), blood biochemical parameters (lipid profiles, liver and kidney function markers), and inflammatory cytokines (IL-6, TNF-α). Additionally, fecal SCFAs and microbiota composition were analyzed to explore microbial and functional changes associated with UA881 supplementation.

This study, therefore, provides an integrated evaluation of the clinical, biochemical, and microbiological effects of UA881 in overweight adults. By combining metabolic assessments with next-generation sequencing (NGS)-based microbiota profiling and SCFA analysis, we aimed to provide a comprehensive overview of how UA881 supplementation impacts host–microbiota interactions. The results of this trial are expected to contribute to the growing body of evidence supporting probiotics as potential adjunctive interventions for metabolic health, while addressing the need for strain-specific, mechanistic data in human populations.

## 2. Materials and Methods

### 2.1. Participant Enrollment

This study was conducted as a randomized, double-blind, placebo-controlled clinical trial. The study protocol was reviewed and approved by the Institutional Review Board of Chung Shan Medical University Hospital (IRB No. CS2-23191) and was performed in accordance with the principles of the Declaration of Helsinki. The trial was registered at ClinicalTrials.gov (Identifier: NCT06554314). A total of 73 volunteers aged 20–65 years were initially screened. Eligible participants were overweight adults with a body mass index (BMI) between 25 and 27 kg/m^2^ and excessive body fat (≥25% in men or ≥30% in women). Additional inclusion criteria included the ability to understand the informed consent document, willingness to comply with dietary restrictions, and agreement to provide blood, urine, and stool samples during the study period. Exclusion criteria included known milk allergy, active cancer or cardiac disease under treatment, concurrent medications that might interfere with the study, systemic infections requiring antibiotics, and current use of probiotic supplements. Participants who were receiving medications with potential metabolic effects were excluded according to the predefined exclusion criteria. Specifically, individuals using antihypertensive, antidiabetic, lipid-lowering, or other metabolism-related medications were not eligible for enrollment; therefore, no participants with diagnosed metabolic comorbidities requiring pharmacological treatment were included in this study. Participants who met all eligibility criteria were randomly assigned in a 1:1 ratio to receive either UA881 probiotics or placebo. Randomization was performed using a computer-generated covariate-adaptive randomization procedure, with stratification by gender, age, and BMI to minimize potential confounding effects. Allocation was conducted by an independent study staff member not involved in participant recruitment, intervention administration, or outcome assessment, ensuring allocation concealment. Both participants and investigators were blinded to group assignments throughout the study period. No post-trial intervention was provided to participants in the placebo group after completion of the study. Participants identified as having potential metabolic health risks based on study assessments were advised to seek further medical evaluation as appropriate.

Of the 73 individuals screened, 23 were excluded due to not meeting inclusion criteria (*n* = 6), personal withdrawal (*n* = 15), or comorbid diseases (*n* = 2). Fifty participants met all criteria and were randomized into two groups: probiotics (*n* = 25) or placebo (*n* = 25). Participants consumed one capsule daily for 28 days, and all completed the trial without loss to follow-up.

### 2.2. UA881 Probiotics and Placebo Capsules Administration

Participants were instructed to take the assigned UA881 probiotic or placebo preparation once daily in the morning under fasting conditions for 28 consecutive days. The 28-day intervention period was selected because probiotic supplementation has been reported to induce measurable effects on gut microbiota and host metabolism within approximately 28 days [21]. The probiotic and placebo products were identical in appearance and packaging to ensure the double-blind design. Both preparations were produced and quality-controlled by NUTRAREX BIO-TECH (Taichung, Taiwan). The detailed composition of UA881 probiotics and placebo is provided in Table 1.

### 2.3. Body Characteristics Assessment

Anthropometric and metabolic-related parameters were assessed at baseline (day 0) and after the 28-day intervention. The measurements included body weight, body mass index (BMI), body fat percentage (BFP), body fat mass (BFM), visceral fat level (VFL), waist and hip circumferences, systolic, diastolic blood pressure and pulse. These indicators were selected because they are closely associated with metabolic disorders such as overweight, insulin resistance, and dyslipidemia. Body composition was measured under standardized conditions using a bioelectrical impedance analyzer (InBody 270, Biospace Co., Ltd., Seoul, Republic of Korea). All anthropometric and bioimpedance assessments were performed by the same trained investigator to minimize inter-observer variability. Waist and hip circumferences were measured using a flexible tape measure. Blood pressure was assessed using an automated sphygmomanometer (DS-G10J, NISSEI, Hamamatsu, Japan) after participants were seated at rest.

During the intervention period, participants were instructed to maintain their usual lifestyle habits, including dietary patterns and physical activity levels. Those with pre-existing exercise routines were asked to keep the same frequency and intensity throughout the study. These assessments provided key outcomes for evaluating the metabolic effects of UA881 probiotic supplementation compared with placebo.

### 2.4. Blood Sampling and Serum Separation

Peripheral blood samples were collected by venipuncture of the cubital vein at baseline (day 0) and after 28 days of intervention following an overnight fast of at least 8 h. Approximately 15 mL of venous blood was obtained from each participant at each time point. Samples were collected into K_2_EDTA anticoagulant tubes (4 mL, BD Vacutainer, Becton, Dickinson and Company, Franklin Lakes, NJ, USA) and serum separator tubes containing clot activator and gel (8.5 mL, BD Vacutainer, Plymouth, UK). After clotting, samples were centrifuged at 3000× g for 15 min, and the resulting serum was carefully separated. Aliquots were stored in sterile microcentrifuge tubes at −80 °C until subsequent analyses.

### 2.5. Serum Parameters Assessment

Serum biochemical analyses were performed using an automated clinical chemistry analyzer (cobas e801, Roche Diagnostics, IN, USA). The parameters measured included blood urea nitrogen (BUN), creatinine, aspartate aminotransferase (AST), alanine aminotransferase (ALT), uric acid (UA), triglycerides (TG), total cholesterol (TC), high-density lipoprotein cholesterol (HDL-C), low-density lipoprotein cholesterol (LDL-C), fasting glucose (Glucose-AC), and vitamin B12. These indicators were selected to evaluate kidney and liver function, lipid and glucose metabolism, as well as micronutrient status in participants receiving UA881 probiotics or placebo.

Serum levels of tumor necrosis factor-α (TNF-α) and interleukin-6 (IL-6) were measured using commercially available enzyme-linked immunosorbent assay (ELISA) kits according to the manufacturers’ instructions (TNF-α: Elabscience, Houston, TX, USA; Cat. No. E-EL-H0109-96T; IL-6: Elabscience, Houston, TX, USA; Cat. No. E-EL-H6156-96T).

### 2.6. Fecal Short-Chain Fatty Acid (SCFA) Assessment

Fecal samples for short-chain fatty acid (SCFA) analysis were collected on a voluntary basis. Among participants who completed the intervention, complete paired fecal samples at baseline (day 0) and day 28 were obtained from 20 participants per group and were included in the SCFA analysis. Stool samples were analyzed for acetic acid, propanoic acid, butanoic acid, isobutanoic acid, and pentanoic acid concentrations. All analyses were conducted by the Health Technology Center, Chung Shan Medical University (Taichung, Taiwan).

For sample pretreatment, approximately 0.5 g of feces was homogenized with 5 mL of deionized water for 2 min, followed by centrifugation (7000 rpm, 25 °C, 5 min). The supernatant was filtered through a 0.45 μm polytetrafluoroethylene (PTFE) membrane, and 1 mL of the filtrate was mixed with the internal standard (isocaproic acid) and 100 μL of 50% (*v*/*v*) sulfuric acid. Subsequently, 1 mL of ether was added, homogenized for 30 s, and centrifuged (4000 rpm, 25 °C, 2 min). The upper layer was then subjected to gas chromatography analysis.

SCFA quantification was performed using gas chromatography with flame ionization detection (GC-FID, Agilent 6890A, Agilent Technologies, Santa Clara, CA, USA) with the following conditions: injection volume, 2 μL; injector temperature, 230 °C; column, DB-FFAP (0.25 mm I.D. × 30 m); detector temperature, 230 °C; carrier gas, nitrogen. SCFAs were selected as key endpoints because they are critical microbial metabolites linked to host energy metabolism, gut barrier integrity, and systemic inflammation in metabolic disorders.

### 2.7. Microbiota NGS and Analysis

Gut microbiota analysis was performed using fecal samples collected on a voluntary basis. Complete paired stool samples at baseline and post-intervention were available from 20 participants per group, and these samples were used for 16S rRNA gene sequencing and subsequent microbiota analysis. Fecal nucleic acid extraction, library preparation, and sequencing were conducted at the Center for Aging and Disease Prevention Research, Fooyin University (Kaohsiung, Taiwan).

#### 2.7.1. Fecal DNA Extraction

Microbial DNA was extracted from stool samples using the QIAamp Fast DNA Stool Mini Kit (QIAGEN, Hilden, Germany) according to the manufacturer’s instructions. Briefly, samples were centrifuged at 13,200 rpm for 10 min to remove the preservation solution, resuspended in Inhibit buffer, and processed sequentially with the kit reagents. DNA was purified using QIAamp spin columns, eluted with preheated elution buffer, and quantified using a NanoDrop 2000 spectrophotometer (Thermo Fisher Scientific, Wilmington, DE, USA) to determine DNA concentration and purity.

#### 2.7.2. Library Construction and Sequencing

16S rRNA gene libraries were prepared following the Illumina 16S Metagenomic Sequencing Library Preparation protocol. The V3–V4 regions of the bacterial 16S rRNA gene were amplified using region-specific primers, followed by PCR clean-up and index PCR to attach Illumina adapters. After a second clean-up step, library quality and fragment size distribution were validated using a Fragment Analyzer (Agilent, Santa Clara, CA, USA), and library concentrations were quantified with a Qubit 3.0 Fluorometer (Thermo Fisher Scientific, Waltham, MA, USA). Libraries were subsequently denatured with NaOH and sequenced on an Illumina MiSeq platform (Illumina, San Diego, CA, USA) using the MiSeq Reagent Kit v3 (600 cycles), generating paired-end reads. Sequencing quality criteria were set at Q30 ≥ 80%, with a minimum sequencing depth of 100,000 reads per sample.

Raw sequencing reads were subjected to quality control prior to downstream analysis. Paired-end reads were merged and filtered to remove low-quality sequences, primer and adapter contamination, and chimeric reads. High-quality reads were clustered into operational taxonomic units (OTUs) at 97% sequence similarity using a standard OTU-based pipeline. Taxonomic assignment was performed using the SILVA ribosomal RNA gene database (release 138), with classification applied from the phylum to genus levels. OTUs with extremely low abundance or low prevalence across samples were filtered out to reduce background noise. To account for differences in sequencing depth among samples, data were normalized by rarefaction to an even sequencing depth prior to diversity analysis.

Alpha diversity indices, including observed OTUs, ACE, Chao1, and Shannon index, were calculated based on the rarefied OTU table. Relative abundance data were used for taxonomic profiling and visualization at the phylum and genus levels, including heatmap generation using log-transformed relative abundance values. Phylum-level composition and *Firmicutes*-to-*Bacteroidetes* (F/B) ratios were calculated based on relative abundance data. As all samples were processed and sequenced in the same laboratory using identical protocols and within a single sequencing batch, potential batch effects were minimized. Microbiome data were analyzed descriptively, consistent with the exploratory nature of the study.

### 2.8. Statistics

All experimental data are presented as mean ± standard deviation (SD). Statistical analyses and data visualization were performed using GraphPad Prism software (version 10.4.1; GraphPad Software, San Diego, CA, USA). Normality of continuous variables was assessed using the Shapiro–Wilk test. When data met the assumption of normal distribution, parametric tests were applied; when normality was not satisfied, appropriate non-parametric tests were used.

This study was designed as an exploratory, proof-of-concept randomized controlled trial. As limited prior human data were available for UA881 probiotic supplementation, a formal a priori sample size calculation was not performed. The sample size was determined based on feasibility and consistency with previous exploratory probiotic intervention studies.

Serum triglyceride (TG) level was defined as the primary endpoint of the study, as specified in the public trial registry. All other anthropometric measurements, serum biochemical parameters, inflammatory cytokines, fecal short-chain fatty acids, and gut microbiota outcomes were considered secondary endpoints.

Within-group comparisons between baseline (day 0) and post-intervention (day 28) were conducted using two-tailed paired *t*-tests. To further evaluate treatment effects while adjusting for baseline variability, analysis of covariance (ANCOVA) was applied. In this model, the change from baseline to day 28 (Δ value) was used as the dependent variable, baseline measurement was included as a covariate, and treatment group (probiotics vs. placebo) was included as a fixed factor. The interaction between baseline values and treatment group was tested to determine whether the slopes of the regression lines differed significantly between groups. If the interaction was not significant, adjusted group means at the average baseline were compared to assess the main treatment effect. A *p*-value < 0.05 was considered statistically significant.

## 3. Results

### 3.1. Demographic Characteristics

**Figure 1:**
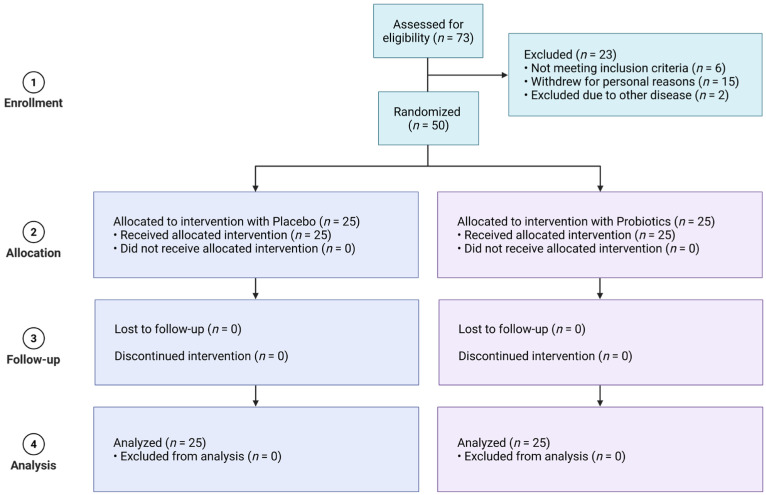
CONSORT flow diagram of enrollment of 73 participants in the clinical trial. Created in BioRender. Chang, V. (2026) https://BioRender.com/140lis9 (accessed date on 24 January 2026).

A total of 73 individuals were initially screened for eligibility. Among them, 23 were excluded due to not meeting the inclusion criteria (*n* = 6), withdrawal for personal reasons (*n* = 15), or the presence of other medical conditions (*n* = 2). The remaining 50 eligible participants were randomized into two groups, with 25 assigned to the UA881 probiotic group and 25 to the placebo group (Figure 1).

The probiotics group consisted of 18 females (72%) and 7 males (28%), with a mean age of 30.76 ± 10.86 years. The placebo group included 16 females (64%) and 9 males (36%), with a mean age of 30.92 ± 12.03 years (Table 2). Baseline demographic characteristics were comparable between the two groups. The distribution of gender did not differ significantly (χ^2^ test, *p* = 0.762), and mean age was similar between groups. These findings indicate that randomization successfully achieved balanced baseline profiles.

### 3.2. Effect of UA881 on Body Characteristics

Body composition and physiological parameters were assessed at baseline and after 28 days of intervention (Table 3). The homogeneity of variances between the placebo and UA881 groups was verified using the Brown–Forsythe test. As shown in Table 3, all parameters yielded non-significant *p*-values (*p* > 0.05), confirming that the assumption of equal variances was met for the data obtained from participants.

In both groups, significant reductions were observed in weight and BMI. Specifically, probiotics supplementation led to highly significant reductions in body weight (73.74 ± 13.49 to 72.68 ± 13.45 kg, *p* < 0.001) and BMI (27.27 ± 3.44 to 26.88 ± 3.54 kg/m^2^, *p* < 0.001). Furthermore, Body Fat Mass (BFM) showed a significant reduction in the probiotics group (24.88 ± 7.69 to 24.30 ± 7.86 kg, *p* = 0.048), while no significant improvement was observed in the placebo group (*p* = 0.208).

While other fat-related indices, such as Body Fat Percentage (BFP), Visceral Fat Level (VFL) and Hip Circumference, showed decreasing trends in the UA881 group, these changes did not reach statistical significance (*p* = 0.520, *p* = 0.059 and *p* = 0.176, respectively). No significant changes were observed in blood pressure or pulse rate for either group during the intervention period.

### 3.3. Serum Parameters Before and After UA881 Intervention

Serum biochemical parameters were evaluated at baseline and after the 28-day intervention (Table 4). The Brown–Forsythe test confirmed the homogeneity of variances for all serum parameters between groups (*p* > 0.05).

In the probiotics group, UA881 supplementation resulted in a significant improvement in lipid metabolism, with significant reductions in the primary endpoint, triglycerides (97.80 ± 46.53 to 81.80 ± 31.66 mg/dL, *p* = 0.034).

A distinct contrast was observed in other metabolic markers. In the placebo group, uric acid (5.20 ± 1.26 to 5.49 ± 1.45 mg/dL, *p* = 0.009) and total cholesterol (177.44 ± 25.32 to 186.56 ± 29.27 mg/dL, *p* = 0.041) significantly increased over the 28-day period, while triglycerides (82.72 ± 36.04 to 88.08 ± 41.22 mg/dL, *p* = 0.096) and LDL-C (108.64 ± 22.97 to 114.76 ± 24.99 mg/dL, *p* = 0.061) showed a modest upward trend. In contrast, the UA881 group demonstrated decreasing trends in uric acid (*p* = 0.213), total cholesterol (*p* = 0.086), and LDL-C (*p* = 0.328), with significant reductions in triglycerides (*p* = 0.034). These findings suggest that UA881 supplementation may help attenuate unfavorable metabolic changes when compared with placebo.

Regarding safety parameters, renal function markers (BUN, Creatinine) showed no significant changes in the probiotics group. ALT levels showed a statistically significant increase in UA881 (*p* = 0.023) groups, although AST levels remained stable. Importantly, despite these statistical increases, the final ALT values in both groups (26.40 ± 37.49 U/L for placebo and 25.40 ± 30.60 U/L for probiotics) remained well within the normal clinical reference range, indicating no clinical hepatotoxicity.

To further evaluate treatment effects while adjusting for baseline variability, analysis of covariance (ANCOVA) was performed using the change from baseline to day 28 (Δ value) as the dependent variable, baseline measurement as a covariate, and treatment group (UA881 probiotics vs. placebo) as a fixed factor. The interaction between baseline values and treatment group was tested to assess whether the regression slopes differed between groups.

**Figure 2:**
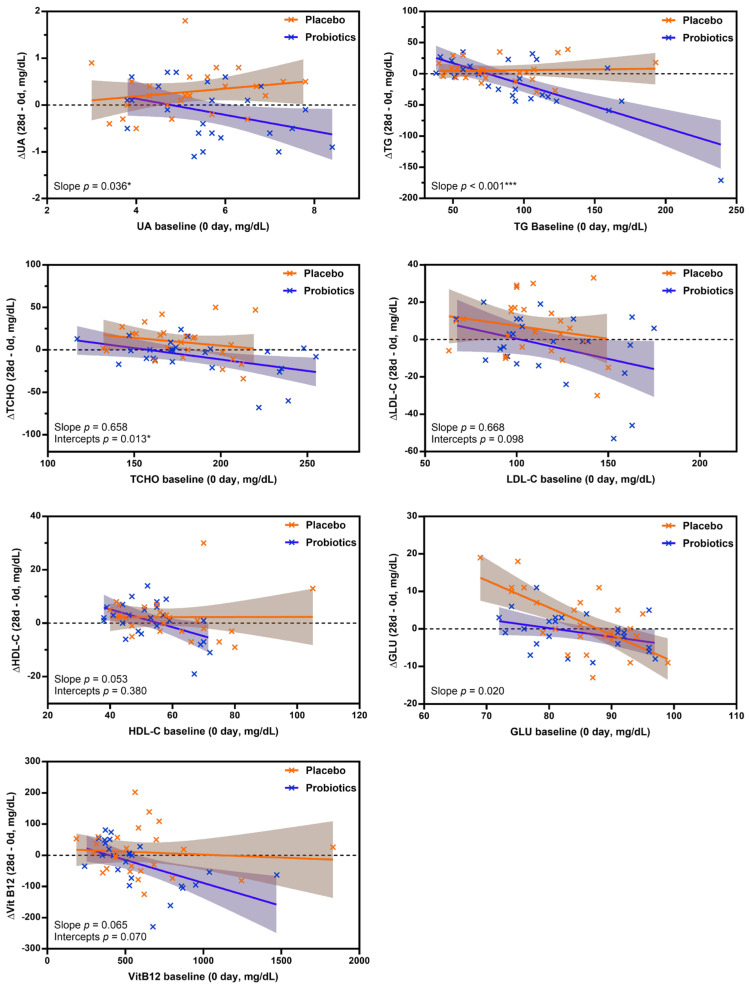
Baseline-adjusted regression analysis of serum parameters following UA881 supplementation.

As shown in Figure 2, serum UA demonstrated a significant treatment × baseline interaction (slope *p* = 0.036), indicating that the relationship between baseline UA levels and subsequent changes differed between the UA881 and placebo groups. This finding suggests a baseline-dependent effect of UA881 supplementation on UA regulation.

A more pronounced effect was observed for TG, for which the regression slopes differed markedly between groups (slope *p* < 0.001). Participants receiving UA881 exhibited a steeper negative slope compared with those receiving placebo, indicating greater TG reductions among individuals with higher baseline TG levels. In contrast, for total cholesterol, the interaction between baseline TCHO and treatment group was not significant (slope *p* = 0.658), indicating comparable regression slopes between groups. However, a significant difference in intercepts was detected (intercept *p* = 0.013), reflecting a consistent group difference in ΔTC after adjustment for baseline values, with the UA881 group showing more favorable changes compared with placebo.

Collectively, these ANCOVA results demonstrate that UA881 supplementation exerted distinct baseline-dependent effects on UA and TG, while also producing a significant group-level difference in total cholesterol independent of baseline TCHO.

Analysis of covariance (ANCOVA) was performed using the change from baseline (Δ value) as the dependent variable, baseline value as a covariate, and treatment group (UA881 vs. placebo) as a fixed factor. Interaction terms between baseline values and treatment group were tested to evaluate differences in regression slopes between groups. When the interaction term was significant, slope differences are reported; when non-significant, group differences in intercepts are presented. Shaded areas represent 95% confidence intervals. Statistical significance was defined as * (*p* < 0.05) and *** (*p* < 0.001). No correction for multiple comparisons was applied due to the exploratory nature of the analysis. Abbreviations: UA, uric acid; TG, triglycerides; TCHO, total cholesterol; LDL-C, low-density lipoprotein cholesterol; HDL-C, high-density lipoprotein cholesterol; Vit B12, Vitamin B12.

### 3.4. Proinflammatory Cytokines Assessment

**Figure 3:**
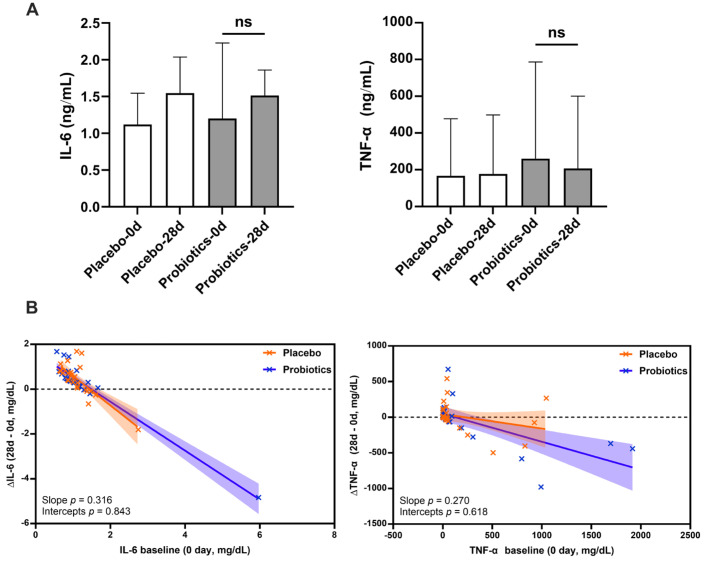
Serum proinflammatory cytokine levels after UA881 supplementation. Serum levels of inflammatory cytokines after 28 days of UA881 or placebo administration. (**A**) Interleukin-6 (IL-6) and (**B**) tumor necrosis factor-α (TNF-α) were measured by ELISA at baseline and day 28. Data are presented as mean ± SD. Within-group comparisons were analyzed using paired two-tailed *t*-tests. Between-group differences were further assessed using ANCOVA with baseline values as covariates. No statistically significant differences were observed. A *p*-value < 0.05 was considered statistically significant. No adjustment for multiple testing was applied. Abbreviations: ns, non-significant.

As probiotic supplementation has been suggested to potentially modulate immune responses [23], we evaluated whether UA881 treatment induced systemic proinflammatory activity. Serum levels of tumor necrosis factor-α (TNF-α) and interleukin-6 (IL-6) were measured in all participants at baseline and after 28 days of intervention using ELISA. As shown in Figure 3A,B, no significant differences were observed in IL-6 or TNF-α concentrations between the probiotics and placebo groups after the intervention. Furthermore, ANCOVA analysis using baseline-adjusted regression confirmed that changes in cytokine levels did not differ significantly between groups. These findings indicate that UA881 supplementation did not provoke a systemic proinflammatory response during the 28-day intervention.

### 3.5. Effects of UA881 on Fecal Short-Chain Fatty Acids (SCFAs)

**Figure 4:**
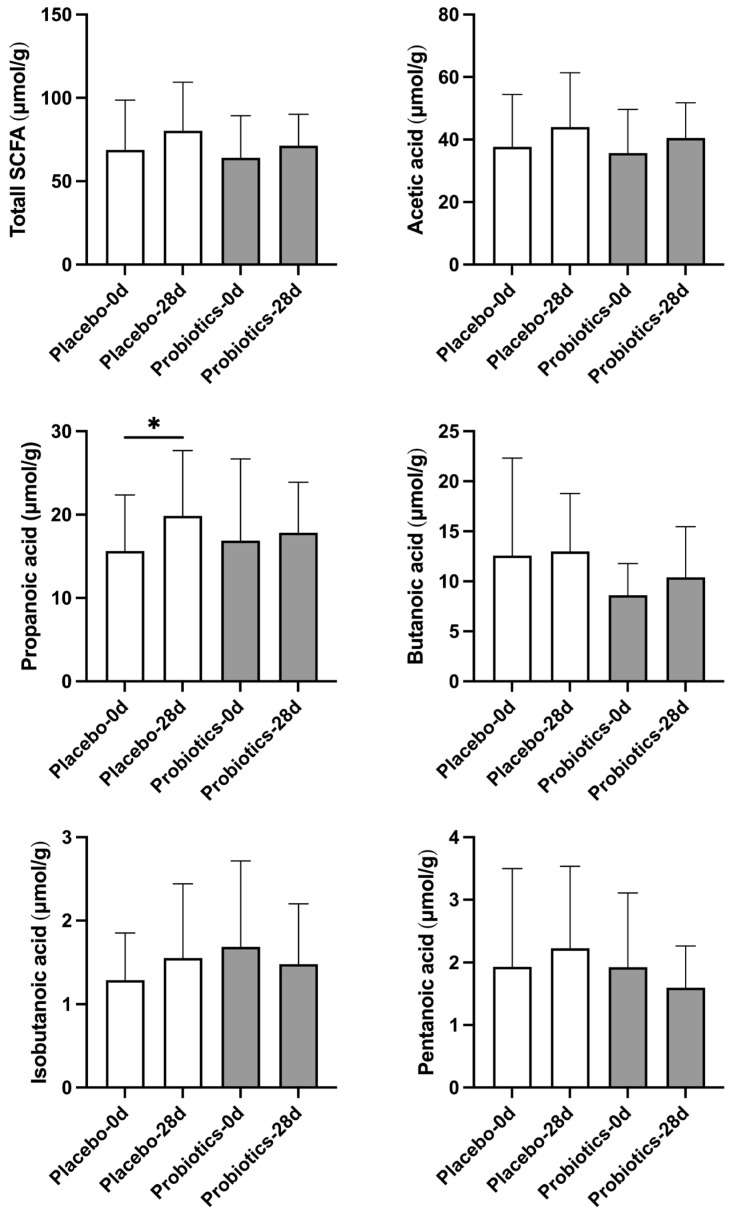
Fecal short-chain fatty acid (SCFA) levels before and after UA881 supplementation. Total SCFAs and individual SCFAs, including acetic acid, propionic acid, and butyric acid, were quantified at baseline and day 28. Data are shown as mean ± SD. Statistical significance was defined as * *p* < 0.05. Within-group comparisons were performed using paired two-tailed *t*-tests. No correction for multiple comparisons was applied due to the exploratory design.

Short-chain fatty acids (SCFAs), including acetic acid, propionic acid, butyric acid, isobutyric acid, and pentanoic acid, are important microbial metabolites that contribute to host energy metabolism, gut barrier function, and regulation of inflammation [24]. To assess the effect of UA881 supplementation on SCFA production, fecal SCFAs were analyzed at baseline and after the 28-day intervention (Figure 4).

In the probiotics group, butanoic acid levels showed an increasing trend following the 28-day UA881 intervention; however, this change did not reach statistical significance after reanalysis using a two-tailed test. Similarly, total SCFAs, acetic acid, and propanoic acid exhibited modest upward trends after UA881 supplementation without achieving statistical significance. In contrast, isobutyric acid and pentanoic acid levels remained relatively stable throughout the intervention period. In the placebo group, propanoic acid levels increased significantly over the intervention period (*p* < 0.05), while other SCFA levels remained unchanged. These findings indicate that UA881 supplementation may be associated with a tendency toward increased butanoic acid production, a metabolite relevant to gut health, distinct from the overall pattern observed in the placebo group.

### 3.6. Alpha Diversity of Gut Microbiota

**Figure 5:**
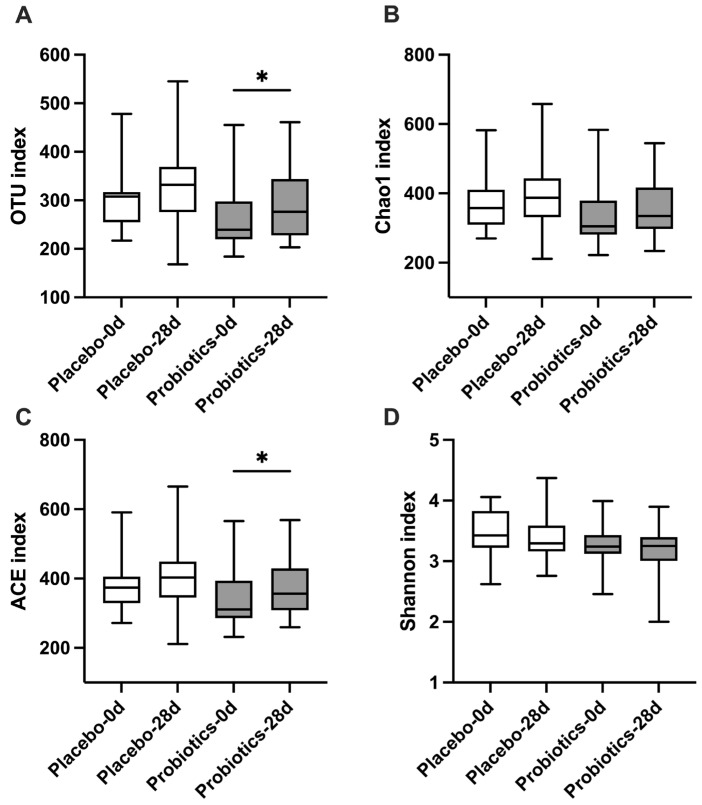
Alpha diversity indices of gut microbiota before and after UA881 supplementation. Effects of UA881 supplementation on gut microbiota alpha diversity. Alpha diversity indices were assessed using (**A**) observed operational taxonomic units (OTUs), (**B**) Chao1 richness estimator, (**C**) ACE index, and (**D**) Shannon diversity index. Data are presented as box-and-whisker plots. Within-group comparisons were analyzed using two-tailed *t*-tests or non-parametric tests where appropriate, based on data distribution. Statistical significance was defined as * *p* < 0.05. No multiple-comparison correction was applied, as these analyses were considered exploratory.

To assess the effect of UA881 supplementation on gut microbial diversity, alpha diversity indices were calculated at baseline and after 28 days of intervention (Figure 5). Compared with the placebo group, the probiotics group demonstrated significant increases in observed OTUs and ACE index, indicating enhanced microbial richness following UA881 treatment. In addition, modest but non-significant increases were observed in Chao1 and Shannon indices, suggesting a trend toward improved community richness and diversity. Together, these results indicate that UA881 supplementation may promote a richer and more diverse gut microbiota within the 28-day intervention period.

### 3.7. Gut Microbiota Composition at the Phylum Level

**Figure 6:**
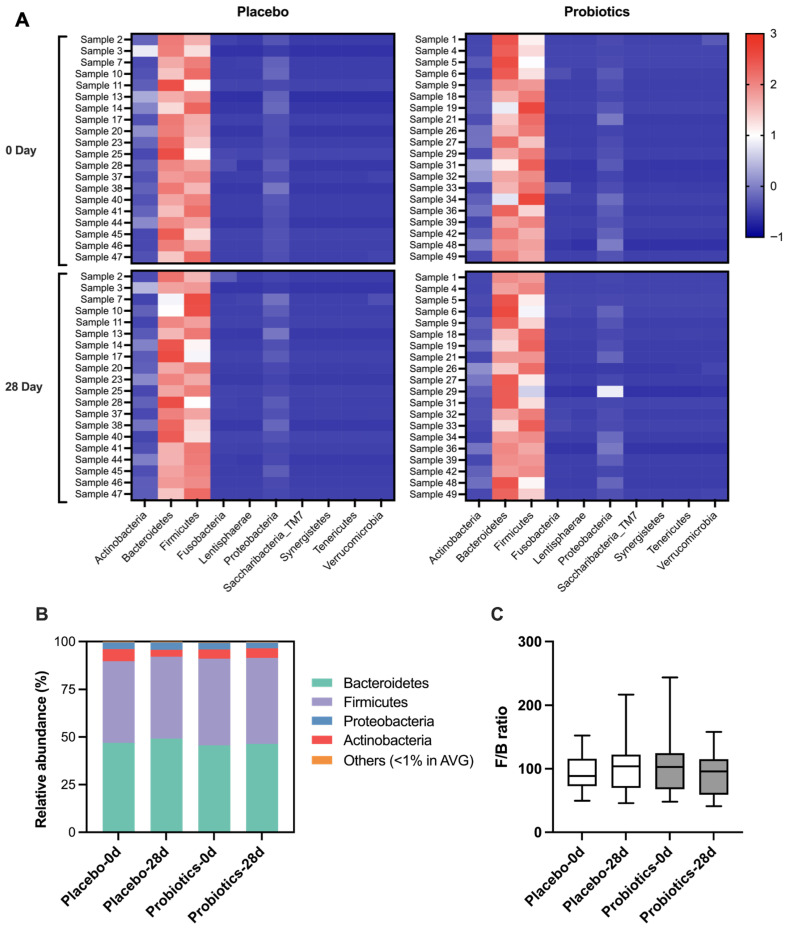
Gut microbiota composition at the phylum level after UA881 supplementation. (**A**) Heatmap of log-transformed read ratios showing phylum-level relative abundance across participants in the probiotics and placebo groups. (**B**) Stacked bar charts of relative abundance ratios for dominant phyla at baseline and after 28 days of intervention. (**C**) *Firmicutes*-to-*Bacteroidetes* (F/B) ratio in the probiotics and placebo groups before and after intervention, presented as box-and-whisker plots showing the median, interquartile range, and minimum to maximum values. Alternating background colors (white and gray) are used for visual distinction between treatment groups.

Phylum-level microbiota composition was examined to assess the impact of UA881 supplementation (Figure 6). The heatmap (Figure 6A), constructed from log-transformed read ratios, showed that *Firmicutes* and *Bacteroidetes* were the dominant phyla across participants, with *Actinobacteria*, *Proteobacteria*, and *Verrucomicrobia* present at lower relative abundances. The distribution patterns demonstrated high inter-individual variability, but no clear group-level shifts were evident after intervention.

Relative abundance data are further illustrated by stacked bar charts (Figure 6B). The overall proportions of *Firmicutes* and *Bacteroidetes* remained predominant in both probiotics and placebo groups at baseline and after 28 days, with only minor differences observed.

The *Firmicutes*-to-*Bacteroidetes* (F/B) ratio was also calculated (Figure 6C). While individual differences were apparent, no statistically significant changes in the F/B ratio were detected between probiotics and placebo groups throughout the study period.

Collectively, these phylum-level analyses indicate that UA881 supplementation did not significantly alter the dominant gut microbiota composition within the 28-day intervention period.

### 3.8. Gut Microbiota Composition at the Genus Level

**Figure 7:**
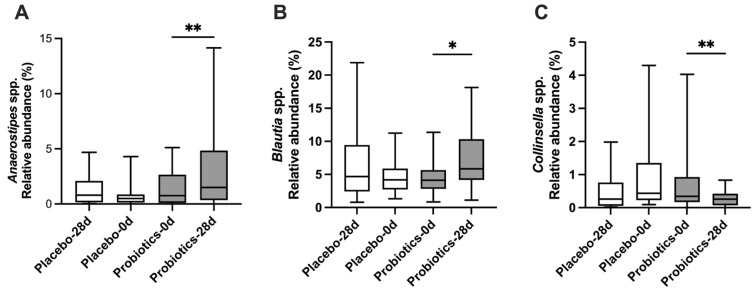
Genus-level changes in gut microbiota after UA881 supplementation. (**A**) *Anaerostipes* spp., (**B**) *Blautia* spp., and (**C**) *Collinsella* spp. abundances before and after 28 days of intervention in probiotics and placebo groups. Data are presented as box-and-whisker plots showing the median, interquartile range, and minimum to maximum values. * *p* < 0.05 and ** *p* < 0.01 was considered statistically significant.

Because no major differences were detected at the phylum level, we further analyzed microbiota composition at the genus level (Figure 7). Notable changes were observed following UA881 supplementation. Beneficial genera, including *Anaerostipes* spp. (Figure 7A) and *Blautia* spp. (Figure 7B), showed significant increases after 28 days of UA881 treatment compared with baseline. In contrast, *Collinsella* spp., a genus often associated with adverse metabolic outcomes, exhibited a significant decrease in abundance in the probiotics group (Figure 7C). All results are presented as box-and-whisker plots, showing the median, interquartile range, and minimum to maximum values. These findings suggest that UA881 supplementation may selectively enhance beneficial commensals while suppressing potentially harmful taxa at the genus level.

## 4. Discussion

In this randomized, placebo-controlled clinical trial, we evaluated the metabolic and microbiota-related effects of UA881 probiotic supplementation in overweight adults. After the 28-day intervention, significant reductions in body weight and BMI were observed in both groups; however, the magnitude of improvement was more pronounced in the probiotics (UA881) group. Regarding serum biochemistry, UA881 supplementation demonstrated more favorable effects compared with placebo. The placebo group showed increases in triglycerides, uric acid, total cholesterol, and LDL-C, suggesting a gradual worsening of metabolic indicators. In contrast, the UA881 group exhibited significant reductions in triglycerides (Primary Endpoint), while other lipid-related markers remained stable throughout the intervention. Furthermore, UA881 specifically enhanced the production of fecal butanoic acid and modulated gut microbiota composition, characterized by increases in alpha diversity (Observed OTUs and ACE) and the enrichment of beneficial genera such as *Anaerostipes* and *Blautia*. Collectively, these findings support our hypothesis that UA881 supplementation may improve metabolic status through beneficial modulation of gut microbiota and associated metabolites.

Our findings regarding weight loss align with previous studies suggesting that probiotics can modulate energy metabolism and body composition [25,26,27]. The significant reductions in body weight and BMI observed in both the UA881 and placebo groups may, in part, reflect a Hawthorne effect [28], whereby participants modify their behavior simply due to awareness of being observed in a clinical study. Increased attention to dietary habits, heightened health awareness, and improved adherence to lifestyle recommendations during the intervention period may have contributed to modest weight reduction in both groups. Besides, maltodextrin, although commonly used as a placebo matrix, is a digestible carbohydrate and cannot be regarded as completely biologically inert under all conditions. Therefore, a mild caloric or fermentative effect on host metabolism and gut microbial activity in the placebo group cannot be entirely excluded. Such effects are commonly reported in short-term nutritional and lifestyle intervention trials. Importantly, however, the magnitude of weight and BMI reduction was more pronounced in the UA881 group than in the placebo group, supporting a potential additive effect of UA881 supplementation beyond study participation–related behavioral changes. Therefore, the difference in lipid profiles between groups highlights a potential protective role of UA881. While previous trials have reported varying degrees of lipid-lowering effects depending on the strain [29,30], our data suggest that UA881 may mitigate metabolic dysregulation.

In this non-interventional trial, participants were instructed to maintain their habitual diet, physical activity, and daily lifestyle throughout the study period. Under these conditions, deterioration trends in some metabolic parameters observed in the placebo group may reflect normal physiological variability rather than effects attributable to the study protocol. As shown in Table 4, the placebo group exhibited upward trends in several metabolic disorder–related parameters, including TG, UA, TCHO, and LDL-C, with UA and TCHO showing statistically significant increases. In contrast, participants receiving UA881 supplementation maintained stable levels or demonstrated downward trends in these parameters, although only the reduction in TG reached statistical significance. These contrasting patterns between groups underscore a clear between-group difference and suggest that UA881 may help stabilize or improve metabolic abnormalities in overweight individuals.

Regarding safety, UA881 appeared well-tolerated. We observed no significant elevation in systemic inflammatory cytokines (IL-6 and TNF-α). Although a statistical increase in ALT was noted in the probiotics group, values remained well within the normal clinical reference range, indicating the absence of clinical hepatotoxicity.

In addition to the changes observed in the UA881 group, a significant increase in fecal propanoic acid was also detected within the placebo group. As no active probiotic strain was administered, this finding is unlikely to reflect a treatment-specific effect. Fecal propionate levels are known to be influenced by short-term variations in habitual diet, particularly fermentable carbohydrate and dietary fiber intake, as well as by intrinsic temporal fluctuations of the gut microbiota. Because participants were instructed to maintain their usual lifestyle without dietary standardization, such within-group temporal changes may occur independently of intervention. Notably, this increase did not translate into a significant between-group difference, suggesting that the observed propanoic acid elevation represents natural variability rather than a placebo-driven biological effect.

Mechanistically, fecal short-chain fatty acid analysis revealed an increasing trend in butyrate levels following UA881 supplementation, although this change did not reach statistical significance after reanalysis using two-tailed tests. Despite the absence of statistical significance, the observed trend remains biologically relevant, as butyrate is a well-established microbial metabolite involved in host lipid and energy metabolism. Previous studies have shown that butyrate can activate AMP-activated protein kinase (AMPK), suppress hepatic lipogenesis, enhance fatty acid oxidation, and modulate cholesterol homeostasis through regulation of lipid-related enzymes, including acetyl-CoA carboxylase and HMG-CoA reductase [31,32]. In addition, butyrate has been reported to influence bile acid metabolism and intestinal lipid absorption, which may collectively contribute to improved circulating lipid profiles [33]. Although a causal relationship cannot be established in the current study, the concurrent trend toward increased butyrate levels and the observed reductions in serum triglycerides and total cholesterol support a plausible link between UA881-induced microbial metabolic activity and host lipid regulation.

Besides, the metabolic improvements associated with UA881 appear to be linked to specific shifts in the gut microbiota and its metabolic output. The observed increase in alpha diversity supports the notion that a richer microbiome is associated with better metabolic health [19,20]. Notably, UA881 supplementation specifically enriched *Anaerostipes* and *Blautia*. Both genera are well-characterized butyrate producers, a finding that is strongly corroborated by our SCFA analysis, which showed a significant increase in fecal butanoic acid exclusively in the probiotics group. In contrast, the placebo group showed an increase in propanoic acids but not butanoic acid. Butyrate is crucial for maintaining gut barrier integrity and exerting anti-inflammatory effects [19,34,35], suggesting that the *Anaerostipes/Blautia*-driven increase in butyrate may be a key pathway through which UA881 improves host metabolism. Additionally, the significant reduction in *Collinsella*, a genus frequently linked to insulin resistance and pro-inflammatory states [34], further supports the beneficial remodeling of the gut ecosystem by UA881.

Several limitations should be noted. First, the sample size was relatively small (*n* = 25 per group), which may limit statistical power for some secondary outcomes. Second, the intervention period was short (28 days), and no post-intervention follow-up assessments were conducted; therefore, the durability of the observed effects over time could not be evaluated. Longer trials are needed to confirm sustained benefits and long-term safety. Third, glycemic control–related markers, such as HbA1c and insulin, were not assessed in the present study; inclusion of these parameters in future studies would allow a more comprehensive evaluation of glucose metabolism and insulin sensitivity in overweight individuals. Fourth, the analysis was limited to 16S rRNA sequencing, which restricts taxonomic resolution to the genus level and does not capture functional gene profiles. Fifth, although baseline covariates were adjusted for, strict control of diet and physical activity was not feasible in this free-living cohort, potentially contributing to the variability observed in both groups. Finally, although participants were instructed to maintain their usual lifestyle habits, including physical activity, exercise was not quantitatively monitored or included as a covariate, which may have contributed to inter-individual variability.

An additional limitation of this study is that the inclusion criteria were restricted to individuals with a BMI of 25–27 kg/m^2^, representing a mildly overweight population rather than individuals with obesity. Therefore, the findings may not be generalizable to populations with more severe obesity or obesity-related metabolic complications. Future studies, including a broader BMI range, are warranted to confirm the applicability of UA881 supplementation across different degrees of adiposity.

Future research should validate these findings in larger cohorts with longer follow-up periods. Integration of metagenomic sequencing and metabolomic profiling would provide deeper insights into the functional mechanisms of UA881, including strain-specific contributions and host–microbe interactions. Additionally, exploring the effects of UA881 in populations with established metabolic syndrome or dyslipidemia would clarify its therapeutic potential beyond preventive applications.

## 5. Conclusions

In summary, UA881 supplementation for 28 days resulted in improvements in body weight, BMI and lipid-related parameters, accompanied by favorable modulation of gut microbiota composition at the genus level, without inducing systemic inflammation. Although phylum-level distributions remained unchanged, increases in alpha diversity and enrichment of beneficial taxa support the role of UA881 in promoting a healthier gut environment. These findings provide preliminary evidence that UA881 may improve metabolic status and warrant further investigation in larger and longer clinical studies.

## Figures and Tables

**Table 1:** The components of the administered capsule.

	Main Ingredient	Dosage Regimen ^†^
Probiotic capsule	*Lactobacillus helveticus* (UA881) and maltodextrin (excipient)	5 × 10^9^ CFU/capsule, 1 capsule/day for 28 days
Placebo capsule	Maltodextrin	1 capsule/day for 28 days

^†^ The selected dose (5 × 10^9^ CFU/day) was based on commonly used probiotic doses (10^9^–10^10^ CFU/day) reported in clinical studies and was considered appropriate for this exploratory trial [22]. CFU, colony-forming unit.

**Table 2:** Demographics between participants assigned to probiotics group and placebo group.

Variable	Probiotic Group (*n* = 25)	Placebo Group (*n* = 25)	*p* Value ^†^
Gender, *n* (%)			
Female	18 (72)	16 (64)	0.762 ^†^
Male	7 (28)	9 (36)	
Age (years)	30.76 ± 10.86	30.92 ± 12.03	

^†^ Gender distribution was analyzed using the Chi-square test; no significant difference was observed between groups (*p* = 0.762).

**Table 3:** Changes in body characteristics before and after UA881 intervention.

	Placebo (*n* = 25)			Probiotics (*n* = 25)			Homogeneity
	0 Day	28 Days	*P*_1_	0 Day	28 Days	*P*_1_	*P*_2_
Weight (kg)	70.91 ± 12.52	70.34 ± 12.62	0.009 **	73.74 ± 13.49	72.68 ± 13.45	<0.001 ***	0.778
BMI (kg/m^2^)	26.28 ± 4.01	26.08 ± 4.09	0.01 *	27.27 ± 3.44	26.88 ± 3.54	<0.001 ***	0.525
BFP (%)	34.20 ± 7.08	34.09 ± 7.10	0.629	33.75 ± 7.33	33.36 ± 7.35	0.520	0.976
BFM (kg)	24.15 ± 6.84	23.90 ± 7.00	0.208	24.88 ± 7.69	24.30 ± 7.86	0.048 *	0.971
VFL (Level)	10.72 ± 3.76	10.60 ± 3.73	0.581	10.64 ± 4.03	10.28 ± 4.11	0.059	0.980
Waist circumference (cm)	87.74 ± 11.23	87.03 ± 11.12	0.048 *	90.03 ± 7.94	89.04 ± 8.10	0.075	0.702
Hip circumference (cm)	103.20 ± 5.43	102.49 ± 6.02	0.068	103.93 ± 7.79	103.64 ± 7.83	0.176	0.889
Waist-hip ratio	0.85 ± 0.07	0.85 ± 0.07	0.845	0.87 ± 0.06	0.86 ± 0.06	0.168	0.604
SBP (mmHg)	112.76 ± 9.93	111.76 ± 12.23	0.468	114.72 ± 12.19	113.04 ± 11.14	0.246	0.832
DBP (mmHg)	69.68 ± 12.69	69.84 ± 10.12	0.928	68.36 ± 8.51	68.12 ± 10.85	0.568	0.914
Pulse (beats/min)	73.32 ± 12.48	75.16 ± 14.70	0.386	72.56 ± 11.15	69.72 ± 12.46	0.174	0.505

Difference between 0 and 28 days was determined using a paired *t*-test (*P*_1_), and homogeneity of variance between Placebo and Probiotics group was analyzed using Brown–Forsythe test (*P*_2_), representing significant differences as * (*p* < 0.05), ** (*p* < 0.01), and *** (*p* < 0.001). Abbreviations: BMI, body mass index; Abbreviation: BFP, body fat percentage; BFM, body fat mass; VFL, visceral fat level; SBP, systolic blood pressure.; DBP, diastolic blood pressure. The quantitative values were presented as mean ± SD.

**Table 4:** Changes in serum parameters before and after UA881 intervention.

	Placebo (*n* = 25)			Probiotics (*n* = 25)			Homogeneity
	0 Day	28 Days	*P*_1_	0 Day	28 Days	*P*_1_	*P*_2_
Primary Endpoint							
Triglyceride (mg/dL)	82.72 ± 36.04	88.08 ± 41.22	0.096	97.80 ± 46.53	81.80 ± 31.66	0.034 *	0.454
Secondary Outcomes							
Uric acid (mg/dL)	5.20 ± 1.26	5.49 ± 1.45	0.009 **	5.64 ± 1.28	5.49 ± 1.19	0.213	0.688
Total cholesterol (mg/dL)	177.44 ± 25.32	186.56 ± 29.27	0.041 *	186.00 ± 37.01	178.44 ± 32.82	0.086	0.621
HDL-C (mg/dL)	58.44 ± 15.62	60.68 ± 17.49	0.236	52.96 ± 10.43	53.84 ± 9.40	0.550	0.150
LDL-C (mg/dL)	108.64 ± 22.97	114.76 ± 24.99	0.061	118.52 ± 30.44	114.96 ± 29.03	0.328	0.632
Glucose AC (mg/dL)	84.92 ± 7.65	87.04 ± 6.53	0.214	84.84 ± 7.97	83.92 ± 7.66	0.363	0.780
Vitamin B12 (pg/mL)	608.32 ± 335.59	617.96 ± 337.77	0.697	580.88 ± 279.43	554.00 ± 246.63	0.144	0.877
BUN (mg/dL)	11.60 ± 3.57	11.48 ± 3.74	0.852	12.04 ± 2.11	12.76 ± 2.68	0.175	0.458
Creatinine (mg/dL)	0.722 ± 0.11	0.745 ± 0.123	0.097	0.734 ± 0.157	0.760 ± 0.17	0.138	0.818
AST (U/L)	18.56 ± 5.19	28.48 ± 39.12	0.477	20.56 ± 7.67	21.72 ± 10.09	0.345	0.373
ALT (U/L)	17.44 ± 12.02	26.40 ± 37.49	0.100	20.96 ± 24.38	25.40 ± 30.60	0.023 *	0.367

Difference between 0 and 28 days was determined using a paired *t*-test (*P*_1_), and homogeneity of variance between Placebo and Probiotics group was analyzed using Brown–Forsythe test (*P*_2_), representing significant differences as * (*p* < 0.05) and ** (*p* < 0.01). Abbreviations: HDL-C, high-density lipoprotein cholesterol; LDL-C, low-density lipoprotein cholesterol; BUN, blood urea nitrogen; AST, Aspartate Transaminase; ALT, Alanine aminotransferase. The quantitative values were presented as mean ± SD.

## Data Availability

The raw data supporting the conclusions of this article will be made available by the authors on request. Please contact the corresponding author Chin-Lin Hsu (email: clhsu@csmu.edu.tw) for more information.

## Abbreviations

The following abbreviations are used in this manuscript:

SCFAs	Fecal short-chain fatty acids
BMI	Body mass index
NGS	Next-generation sequencing
BFP	Body fat percentage
BFM	Body fat mass
VFL	Visceral fat level
BUN	Blood urea nitrogen
AST	Aspartate aminotransferase
ALT	Alanine aminotransferase
UA	Uric acid
TG	Triglycerides
TC	Total cholesterol
HDL-C	High-density lipoprotein cholesterol
LDL-C	Low-density lipoprotein cholesterol
Glucose-AC	Fasting glucose
